# (5,5′-Dicarboxy­biphenyl-2,2′-dicarboxyl­ato-κ^2^ *O* ^2^,*O* ^2′^)bis­(1,10-phenanthroline-κ^2^ *N*,*N*′)cobalt(II) dihydrate

**DOI:** 10.1107/S160053680801012X

**Published:** 2008-05-03

**Authors:** Ruizhan Chen, Feijun Guo, Fanlei Meng

**Affiliations:** a College of Chemistry, Changchun Normal University, Changchun 130032, People’s Republic of China; b Changchun Institute of Applied Chemistry, Chinese Academy of Sciences, Changchun Center of Mass Spectrometry, Changchun 130022, People’s Republic of China

## Abstract

In the title compound, [Co(C_16_H_8_O_8_)(C_12_H_8_N_2_)_2_]·2H_2_O, the Co atom located on a twofold rotation axis. It is six-coordinated by two O atoms from one 5,5′-dicarboxy­biphenyl-2,2′-dicarboxyl­ate anion and four N atoms from two 1,10-phenanthroline mol­ecules in a distorted octa­hedral environment. The crystal packing is stabilized by O—H⋯O hydrogen bonds.

## Related literature

For related literature, see: Zang *et al.* (2006 ▶); Che *et al.* (2006 ▶); Lehn (1990 ▶).
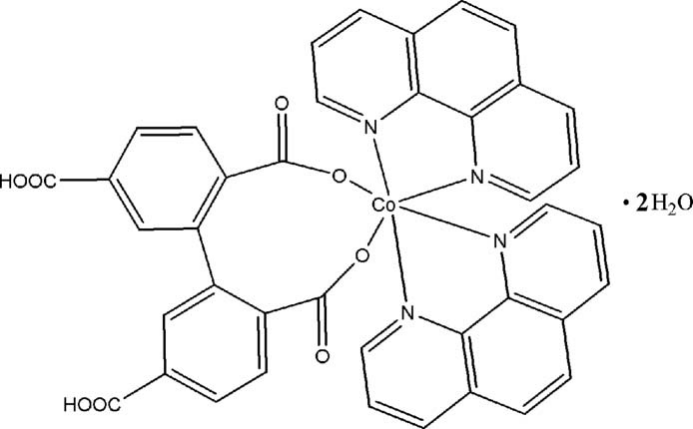


## Experimental

### 

#### Crystal data

[Co(C_16_H_8_O_8_)(C_12_H_8_N_2_)_2_]·2H_2_O
*M* *_r_* = 783.59
Monoclinic, 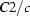

*a* = 16.9272 (14) Å
*b* = 9.4514 (8) Å
*c* = 22.0458 (19) Å
β = 96.056 (1)°
*V* = 3507.3 (5) Å^3^
*Z* = 4
Mo *K*α radiation
μ = 0.56 mm^−1^
*T* = 293 (2) K
0.28 × 0.25 × 0.23 mm

#### Data collection

Bruker APEX CCD area-detector diffractometer
Absorption correction: multi-scan (*SADABS*; Bruker, 1998 ▶) *T*_min_ = 0.852, *T*_max_ = 0.880
9540 measured reflections
3447 independent reflections
2705 reflections with *I* > 2σ(*I*)
*R*_int_ = 0.039

#### Refinement

*R*[*F* ^2^ > 2σ(*F* ^2^)] = 0.047
*wR*(*F* ^2^) = 0.111
*S* = 1.05
3447 reflections
255 parameters
2 restraints
H atoms treated by a mixture of independent and constrained refinement
Δρ_max_ = 0.43 e Å^−3^
Δρ_min_ = −0.21 e Å^−3^

### 

Data collection: *SMART* (Bruker, 1998 ▶); cell refinement: *SAINT* (Bruker, 1998 ▶); data reduction: *SAINT*; program(s) used to solve structure: *SHELXS97* (Sheldrick, 2008 ▶); program(s) used to refine structure: *SHELXL97* (Sheldrick, 2008 ▶); molecular graphics: *SHELXTL* (Sheldrick, 2008 ▶); software used to prepare material for publication: *SHELXTL*.

## Supplementary Material

Crystal structure: contains datablocks global, I. DOI: 10.1107/S160053680801012X/bt2694sup1.cif

Structure factors: contains datablocks I. DOI: 10.1107/S160053680801012X/bt2694Isup2.hkl

Additional supplementary materials:  crystallographic information; 3D view; checkCIF report

## Figures and Tables

**Table 1:** Selected bond lengths (Å)

N1—Co1	2.121 (2)
N2—Co1	2.155 (2)
O1—Co1	2.0865 (16)

**Table 2:** Hydrogen-bond geometry (Å, °)

*D*—H⋯*A*	*D*—H	H⋯*A*	*D*⋯*A*	*D*—H⋯*A*
O1*W*—H1*A*⋯O2	0.890 (10)	1.929 (11)	2.811 (3)	171 (3)
O4—H4⋯O2^i^	0.82	1.74	2.535 (2)	163
O1*W*—H1*B*⋯O3^ii^	0.889 (10)	2.177 (19)	2.934 (3)	143 (2)

Symmetry codes: (i) 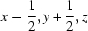; (ii) 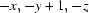.

**Fig. 1.**
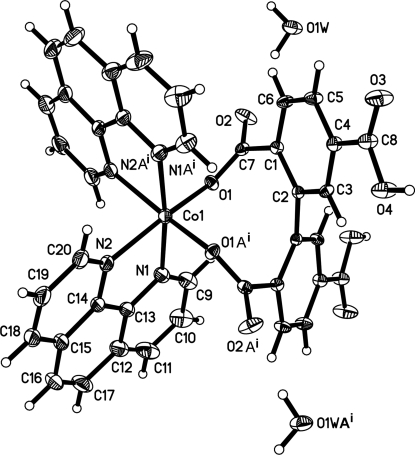
The structure of (I), showing the atomic numbering scheme. Displacement ellipsoids are drawn at the 30% probability level. Symmetry code: (i) -x, y, 0.5 - z.

## supplementary crystallographic information

### Comment

Aromatic polycarboxylate ligands have been extensively employed in the preparation of metal-organic coordination complexes due to their ability to form networks and due to their interesting properties (Lehn, 1990; Che *et al.*, 2006). We selected biphenyl-2,5,2',5'-tetracarboxylic acid (H_4_BPTC) as a bridging ligand, 1,10-phenanthroline as a neutral ligand, and Co^II^ as a metal center, in order to generate a new compound, [Co(H_2_BPTC)(Phen)_2_]^.^2H_2_O, (I), which is reported here.

In compound (I), each Co^II^ atom is six-coordinated by two O atoms from one H_2_BPTC anion and four N atoms from two 1,10-phenanthroline molecules in a distorted octahedral environment (Fig. 1). The bond lengths are all within the normal ranges (Zang *et al.*, 2006). The crystal packing is stabilized by O—H···O hydrogen bonds between carboxylate groups  and water molecules.

### Experimental

A mixture of CoCl_2_^.^2H_2_O (0.1 mmol), biphenyl-2,5,2',5'-tetracarboxylic acid (0.2 mmol), 1,10-phenanthroline (0.2 mmol) and H_2_O(15 ml) in a 25 ml stainless steel reactor with a Teflon liner was heated from 298 to 443 K in 2 h and a constant temperature was maintained at 443 K for 72 h, after which the mixture was cooled to 298 K. Then, pink crystals of were obtained.

### Refinement

The water H-atoms were located from a difference Fourier map, and were refined with distance restraints of O–H = 0.90 Å and *U*_iso_(H) = 1.5*U*_eq_(O). Other H atoms were positioned geometrically (C—H = 0.93 Å and O—H = 0.82 Å) and refined as riding, with *U*_iso_(H) = 1.2*U*_eq_(C) and *U*_iso_(H) = 1.5*U*_eq_(O).

### Crystal data

**Table:**

[Co(C_16_H_8_O_8_)(C_12_H_8_N_2_)_2_]·2H_2_O	*F*_000_ = 1612
*M**_r_* = 783.59	*D*_x_ = 1.484 Mg m^−^^3^
Monoclinic, *C*2/*c*	Mo *K*α radiation λ = 0.71073 Å
Hall symbol: -C 2yc	Cell parameters from 3447 reflections
*a* = 16.9272 (14) Å	θ = 2.0–26.0º
*b* = 9.4514 (8) Å	µ = 0.56 mm^−^^1^
*c* = 22.0458 (19) Å	*T* = 293 (2) K
β = 96.0560 (10)º	Block, pink
*V* = 3507.3 (5) Å^3^	0.28 × 0.25 × 0.23 mm
*Z* = 4	

### Data collection

**Table:**

Bruker APEX CCD area-detector diffractometer	3447 independent reflections
Radiation source: fine-focus sealed tube	2705 reflections with *I* > 2σ(*I*)
Monochromator: graphite	*R*_int_ = 0.039
*T* = 293(2) K	θ_max_ = 26.0º
φ and ω scans	θ_min_ = 1.9º
Absorption correction: multi-scan(SADABS; Bruker, 1998)	*h* = −20→20
*T*_min_ = 0.852, *T*_max_ = 0.880	*k* = −11→10
9540 measured reflections	*l* = −27→22

### Refinement

**Table:**

Refinement on *F*^2^	Secondary atom site location: difference Fourier map
Least-squares matrix: full	Hydrogen site location: inferred from neighbouring sites
*R*[*F*^2^ > 2σ(*F*^2^)] = 0.047	H atoms treated by a mixture of independent and constrained refinement
*wR*(*F*^2^) = 0.111	*w* = 1/[σ^2^(*F*_o_^2^) + (0.0514*P*)^2^ + 0.7022*P*] where *P* = (*F*_o_^2^ + 2*F*_c_^2^)/3
*S* = 1.05	(Δ/σ)_max_ < 0.001
3447 reflections	Δρ_max_ = 0.43 e Å^−^^3^
255 parameters	Δρ_min_ = −0.21 e Å^−^^3^
2 restraints	Extinction correction: none
Primary atom site location: structure-invariant direct methods	

### Special details

**Table:**

Geometry. All e.s.d.'s (except the e.s.d. in the dihedral angle between two l.s. planes) are estimated using the full covariance matrix. The cell e.s.d.'s are taken into account individually in the estimation of e.s.d.'s in distances, angles and torsion angles; correlations between e.s.d.'s in cell parameters are only used when they are defined by crystal symmetry. An approximate (isotropic) treatment of cell e.s.d.'s is used for estimating e.s.d.'s involving l.s. planes.
Refinement. Refinement of *F*^2^ against ALL reflections. The weighted *R*-factor *wR* and goodness of fit *S* are based on *F*^2^, conventional *R*-factors *R* are based on *F*, with *F* set to zero for negative *F*^2^. The threshold expression of *F*^2^ > σ(*F*^2^) is used only for calculating *R*-factors(gt) *etc*. and is not relevant to the choice of reflections for refinement. *R*-factors based on *F*^2^ are statistically about twice as large as those based on *F*, and *R*- factors based on ALL data will be even larger.

### Fractional atomic coordinates and isotropic or equivalent isotropic displacement parameters (Å^2^)

**Table:**

	*x*	*y*	*z*	*U*_iso_*/*U*_eq_	
C1	0.02180 (12)	0.3926 (2)	0.17029 (10)	0.0195 (5)	
C2	−0.01759 (12)	0.4503 (2)	0.21744 (10)	0.0176 (5)	
C3	−0.08951 (13)	0.5192 (2)	0.20193 (10)	0.0210 (5)	
H3	−0.1170	0.5561	0.2327	0.025*	
C4	−0.12130 (13)	0.5340 (3)	0.14147 (11)	0.0240 (5)	
C5	−0.07984 (13)	0.4819 (3)	0.09534 (11)	0.0281 (6)	
H5	−0.0999	0.4935	0.0547	0.034*	
C6	−0.00843 (13)	0.4127 (3)	0.11002 (11)	0.0265 (6)	
H6	0.0197	0.3791	0.0790	0.032*	
C7	0.09258 (13)	0.2983 (2)	0.18419 (11)	0.0227 (5)	
C8	−0.19930 (14)	0.6078 (3)	0.12529 (11)	0.0305 (6)	
C9	0.12655 (16)	0.1382 (3)	0.36037 (13)	0.0419 (7)	
H9	0.1462	0.2070	0.3358	0.050*	
C10	0.16476 (18)	0.1183 (4)	0.41931 (15)	0.0551 (9)	
H10	0.2081	0.1740	0.4335	0.066*	
C11	0.13745 (19)	0.0158 (4)	0.45565 (15)	0.0579 (10)	
H11	0.1632	−0.0008	0.4944	0.069*	
C12	0.07085 (19)	−0.0637 (3)	0.43443 (14)	0.0463 (8)	
C13	0.03635 (17)	−0.0368 (3)	0.37519 (13)	0.0369 (7)	
C14	−0.03385 (18)	−0.1126 (3)	0.35129 (13)	0.0393 (7)	
C15	−0.0675 (2)	−0.2122 (3)	0.38854 (15)	0.0500 (8)	
C16	−0.0295 (3)	−0.2393 (4)	0.44813 (17)	0.0651 (11)	
H16	−0.0508	−0.3072	0.4723	0.078*	
C17	0.0364 (2)	−0.1692 (4)	0.47042 (16)	0.0637 (10)	
H17	0.0598	−0.1893	0.5095	0.076*	
C18	−0.1378 (2)	−0.2783 (3)	0.36436 (17)	0.0612 (10)	
H18	−0.1619	−0.3455	0.3871	0.073*	
C19	−0.1707 (2)	−0.2438 (3)	0.30749 (17)	0.0573 (10)	
H19	−0.2184	−0.2849	0.2915	0.069*	
C20	−0.13227 (18)	−0.1458 (3)	0.27306 (15)	0.0472 (8)	
H20	−0.1551	−0.1239	0.2339	0.057*	
N1	0.06377 (12)	0.0635 (2)	0.33797 (10)	0.0342 (5)	
N2	−0.06511 (14)	−0.0830 (2)	0.29367 (11)	0.0369 (6)	
O1	0.09051 (9)	0.20921 (16)	0.22641 (7)	0.0257 (4)	
O2	0.14953 (10)	0.3116 (2)	0.15270 (8)	0.0443 (5)	
O1W	0.16597 (12)	0.4484 (2)	0.04153 (9)	0.0432 (5)	
O3	−0.22748 (11)	0.6294 (2)	0.07347 (8)	0.0507 (6)	
O4	−0.23287 (10)	0.6461 (2)	0.17334 (8)	0.0475 (6)	
H4	−0.2751	0.6858	0.1626	0.071*	
Co1	0.0000	0.07672 (5)	0.2500	0.02804 (17)	
H1A	0.1647 (16)	0.398 (3)	0.0755 (8)	0.042*	
H1B	0.1857 (15)	0.388 (2)	0.0163 (10)	0.042*	

### Atomic displacement parameters (Å^2^)

**Table:**

	*U*^11^	*U*^22^	*U*^33^	*U*^12^	*U*^13^	*U*^23^
C1	0.0143 (10)	0.0227 (13)	0.0218 (12)	0.0028 (9)	0.0030 (9)	0.0004 (10)
C2	0.0153 (11)	0.0177 (12)	0.0199 (12)	−0.0020 (9)	0.0029 (9)	0.0005 (9)
C3	0.0181 (11)	0.0248 (13)	0.0203 (13)	0.0026 (9)	0.0033 (10)	−0.0037 (10)
C4	0.0193 (12)	0.0288 (14)	0.0238 (13)	0.0063 (10)	0.0016 (10)	0.0001 (10)
C5	0.0236 (12)	0.0418 (16)	0.0180 (13)	0.0087 (11)	−0.0021 (10)	0.0010 (11)
C6	0.0245 (12)	0.0360 (15)	0.0196 (13)	0.0085 (11)	0.0058 (10)	−0.0030 (11)
C7	0.0198 (12)	0.0260 (13)	0.0222 (13)	0.0052 (10)	0.0019 (10)	−0.0028 (10)
C8	0.0227 (12)	0.0443 (17)	0.0242 (14)	0.0110 (11)	0.0020 (11)	0.0005 (12)
C9	0.0319 (15)	0.0500 (18)	0.0444 (18)	0.0084 (14)	0.0072 (13)	0.0125 (14)
C10	0.0368 (16)	0.079 (3)	0.048 (2)	0.0138 (16)	−0.0001 (15)	0.0097 (18)
C11	0.051 (2)	0.081 (3)	0.0416 (19)	0.0279 (19)	0.0070 (16)	0.0244 (18)
C12	0.0533 (19)	0.0457 (19)	0.0425 (18)	0.0193 (15)	0.0168 (15)	0.0117 (15)
C13	0.0483 (17)	0.0286 (15)	0.0371 (17)	0.0160 (13)	0.0195 (14)	0.0069 (12)
C14	0.0587 (19)	0.0238 (15)	0.0404 (17)	0.0087 (13)	0.0283 (15)	0.0025 (12)
C15	0.079 (2)	0.0265 (16)	0.051 (2)	0.0043 (16)	0.0385 (18)	0.0022 (14)
C16	0.106 (3)	0.038 (2)	0.060 (2)	0.007 (2)	0.048 (2)	0.0151 (17)
C17	0.092 (3)	0.055 (2)	0.049 (2)	0.026 (2)	0.029 (2)	0.0256 (18)
C18	0.094 (3)	0.0323 (18)	0.067 (3)	−0.0128 (18)	0.056 (2)	−0.0067 (16)
C19	0.074 (2)	0.0376 (18)	0.068 (2)	−0.0217 (17)	0.043 (2)	−0.0196 (17)
C20	0.060 (2)	0.0317 (16)	0.054 (2)	−0.0110 (15)	0.0279 (16)	−0.0119 (14)
N1	0.0352 (12)	0.0323 (13)	0.0367 (13)	0.0091 (10)	0.0119 (10)	0.0061 (10)
N2	0.0485 (14)	0.0236 (12)	0.0426 (14)	−0.0036 (11)	0.0238 (12)	−0.0048 (10)
O1	0.0231 (9)	0.0249 (9)	0.0294 (10)	0.0044 (7)	0.0038 (7)	0.0069 (8)
O2	0.0297 (10)	0.0664 (14)	0.0402 (12)	0.0286 (9)	0.0196 (9)	0.0277 (10)
O1W	0.0472 (12)	0.0570 (14)	0.0262 (11)	0.0127 (10)	0.0079 (9)	0.0085 (9)
O3	0.0378 (11)	0.0889 (17)	0.0242 (11)	0.0349 (11)	−0.0027 (9)	0.0028 (10)
O4	0.0356 (11)	0.0810 (15)	0.0264 (10)	0.0384 (10)	0.0057 (9)	0.0074 (10)
Co1	0.0308 (3)	0.0225 (3)	0.0323 (3)	0.000	0.0103 (2)	0.000

### Geometric parameters (Å, °)

**Table:**

C1—C6	1.385 (3)	C13—N1	1.367 (3)
C1—C2	1.403 (3)	C13—C14	1.439 (4)
C1—C7	1.499 (3)	C14—N2	1.353 (4)
C2—C3	1.391 (3)	C14—C15	1.408 (4)
C2—C2^i^	1.495 (4)	C15—C18	1.399 (5)
C3—C4	1.391 (3)	C15—C16	1.424 (5)
C3—H3	0.9300	C16—C17	1.345 (5)
C4—C5	1.385 (3)	C16—H16	0.9300
C4—C8	1.503 (3)	C17—H17	0.9300
C5—C6	1.382 (3)	C18—C19	1.357 (5)
C5—H5	0.9300	C18—H18	0.9300
C6—H6	0.9300	C19—C20	1.401 (4)
C7—O2	1.252 (3)	C19—H19	0.9300
C7—O1	1.258 (3)	C20—N2	1.320 (4)
C8—O3	1.208 (3)	C20—H20	0.9300
C8—O4	1.305 (3)	N1—Co1	2.121 (2)
C9—N1	1.327 (3)	N2—Co1	2.155 (2)
C9—C10	1.402 (4)	O1—Co1	2.0865 (16)
C9—H9	0.9300	O1W—H1A	0.890 (10)
C10—C11	1.368 (4)	O1W—H1B	0.889 (10)
C10—H10	0.9300	O4—H4	0.8200
C11—C12	1.394 (5)	Co1—O1^i^	2.0865 (16)
C11—H11	0.9300	Co1—N1^i^	2.121 (2)
C12—C13	1.396 (4)	Co1—N2^i^	2.155 (2)
C12—C17	1.437 (4)		
			
C6—C1—C2	120.1 (2)	C18—C15—C14	117.2 (3)
C6—C1—C7	118.9 (2)	C18—C15—C16	123.7 (3)
C2—C1—C7	120.8 (2)	C14—C15—C16	119.2 (3)
C3—C2—C1	118.1 (2)	C17—C16—C15	121.7 (3)
C3—C2—C2^i^	119.1 (2)	C17—C16—H16	119.2
C1—C2—C2^i^	122.6 (2)	C15—C16—H16	119.2
C2—C3—C4	121.5 (2)	C16—C17—C12	120.7 (3)
C2—C3—H3	119.2	C16—C17—H17	119.7
C4—C3—H3	119.2	C12—C17—H17	119.7
C5—C4—C3	119.5 (2)	C19—C18—C15	119.6 (3)
C5—C4—C8	119.4 (2)	C19—C18—H18	120.2
C3—C4—C8	121.0 (2)	C15—C18—H18	120.2
C6—C5—C4	119.6 (2)	C18—C19—C20	119.4 (3)
C6—C5—H5	120.2	C18—C19—H19	120.3
C4—C5—H5	120.2	C20—C19—H19	120.3
C5—C6—C1	121.0 (2)	N2—C20—C19	122.9 (3)
C5—C6—H6	119.5	N2—C20—H20	118.5
C1—C6—H6	119.5	C19—C20—H20	118.5
O2—C7—O1	124.1 (2)	C9—N1—C13	117.1 (2)
O2—C7—C1	118.2 (2)	C9—N1—Co1	128.26 (19)
O1—C7—C1	117.7 (2)	C13—N1—Co1	114.67 (19)
O3—C8—O4	124.0 (2)	C20—N2—C14	117.9 (3)
O3—C8—C4	123.5 (2)	C20—N2—Co1	128.6 (2)
O4—C8—C4	112.5 (2)	C14—N2—Co1	113.37 (18)
N1—C9—C10	123.2 (3)	C7—O1—Co1	131.64 (15)
N1—C9—H9	118.4	H1A—O1W—H1B	103 (3)
C10—C9—H9	118.4	C8—O4—H4	109.5
C11—C10—C9	119.1 (3)	O1—Co1—O1^i^	106.24 (9)
C11—C10—H10	120.5	O1—Co1—N1^i^	97.10 (7)
C9—C10—H10	120.5	O1^i^—Co1—N1^i^	86.97 (7)
C10—C11—C12	119.8 (3)	O1—Co1—N1	86.97 (7)
C10—C11—H11	120.1	O1^i^—Co1—N1	97.10 (7)
C12—C11—H11	120.1	N1^i^—Co1—N1	173.25 (12)
C11—C12—C13	117.4 (3)	O1—Co1—N2	162.80 (8)
C11—C12—C17	123.4 (3)	O1^i^—Co1—N2	83.42 (7)
C13—C12—C17	119.2 (3)	N1^i^—Co1—N2	97.60 (9)
N1—C13—C12	123.5 (3)	N1—Co1—N2	77.60 (9)
N1—C13—C14	116.4 (3)	O1—Co1—N2^i^	83.42 (7)
C12—C13—C14	120.0 (3)	O1^i^—Co1—N2^i^	162.80 (8)
N2—C14—C15	122.9 (3)	N1^i^—Co1—N2^i^	77.60 (9)
N2—C14—C13	117.8 (2)	N1—Co1—N2^i^	97.60 (8)
C15—C14—C13	119.2 (3)	N2—Co1—N2^i^	91.07 (11)
			
C6—C1—C2—C3	4.1 (3)	C14—C15—C18—C19	−0.7 (4)
C7—C1—C2—C3	−170.5 (2)	C16—C15—C18—C19	178.7 (3)
C6—C1—C2—C2^i^	−171.09 (18)	C15—C18—C19—C20	2.1 (5)
C7—C1—C2—C2^i^	14.4 (3)	C18—C19—C20—N2	−0.8 (5)
C1—C2—C3—C4	−1.4 (3)	C10—C9—N1—C13	0.0 (4)
C2^i^—C2—C3—C4	173.90 (19)	C10—C9—N1—Co1	−178.9 (2)
C2—C3—C4—C5	−1.4 (4)	C12—C13—N1—C9	0.0 (4)
C2—C3—C4—C8	179.6 (2)	C14—C13—N1—C9	177.3 (2)
C3—C4—C5—C6	1.7 (4)	C12—C13—N1—Co1	179.0 (2)
C8—C4—C5—C6	−179.4 (2)	C14—C13—N1—Co1	−3.6 (3)
C4—C5—C6—C1	1.0 (4)	C19—C20—N2—C14	−1.9 (4)
C2—C1—C6—C5	−3.9 (4)	C19—C20—N2—Co1	−176.8 (2)
C7—C1—C6—C5	170.7 (2)	C15—C14—N2—C20	3.4 (4)
C6—C1—C7—O2	45.0 (3)	C13—C14—N2—C20	−175.8 (2)
C2—C1—C7—O2	−140.4 (2)	C15—C14—N2—Co1	179.0 (2)
C6—C1—C7—O1	−134.1 (2)	C13—C14—N2—Co1	−0.1 (3)
C2—C1—C7—O1	40.5 (3)	O2—C7—O1—Co1	−137.2 (2)
C5—C4—C8—O3	−2.7 (4)	C1—C7—O1—Co1	41.8 (3)
C3—C4—C8—O3	176.3 (3)	C7—O1—Co1—O1^i^	−63.12 (19)
C5—C4—C8—O4	177.5 (2)	C7—O1—Co1—N1^i^	25.8 (2)
C3—C4—C8—O4	−3.6 (3)	C7—O1—Co1—N1	−159.6 (2)
N1—C9—C10—C11	1.1 (5)	C7—O1—Co1—N2	174.4 (2)
C9—C10—C11—C12	−2.1 (5)	C7—O1—Co1—N2^i^	102.3 (2)
C10—C11—C12—C13	2.0 (4)	C9—N1—Co1—O1	9.2 (2)
C10—C11—C12—C17	−177.4 (3)	C13—N1—Co1—O1	−169.69 (17)
C11—C12—C13—N1	−0.9 (4)	C9—N1—Co1—O1^i^	−96.8 (2)
C17—C12—C13—N1	178.5 (3)	C13—N1—Co1—O1^i^	84.31 (17)
C11—C12—C13—C14	−178.3 (2)	C9—N1—Co1—N2	−178.4 (2)
C17—C12—C13—C14	1.2 (4)	C13—N1—Co1—N2	2.68 (17)
N1—C13—C14—N2	2.5 (3)	C9—N1—Co1—N2^i^	92.2 (2)
C12—C13—C14—N2	180.0 (2)	C13—N1—Co1—N2^i^	−86.74 (17)
N1—C13—C14—C15	−176.7 (2)	C20—N2—Co1—O1	−159.6 (2)
C12—C13—C14—C15	0.8 (4)	C14—N2—Co1—O1	25.3 (3)
N2—C14—C15—C18	−2.1 (4)	C20—N2—Co1—O1^i^	75.0 (2)
C13—C14—C15—C18	177.0 (2)	C14—N2—Co1—O1^i^	−100.13 (17)
N2—C14—C15—C16	178.4 (3)	C20—N2—Co1—N1^i^	−11.1 (2)
C13—C14—C15—C16	−2.4 (4)	C14—N2—Co1—N1^i^	173.83 (17)
C18—C15—C16—C17	−177.4 (3)	C20—N2—Co1—N1	173.8 (2)
C14—C15—C16—C17	2.1 (5)	C14—N2—Co1—N1	−1.35 (17)
C15—C16—C17—C12	0.0 (5)	C20—N2—Co1—N2^i^	−88.7 (2)
C11—C12—C17—C16	177.8 (3)	C14—N2—Co1—N2^i^	96.20 (19)
C13—C12—C17—C16	−1.6 (5)		

Symmetry codes: (i) −*x*, *y*, −*z*+1/2.

### Hydrogen-bond geometry (Å, °)

**Table:**

*D*—H···*A*	*D*—H	H···*A*	*D*···*A*	*D*—H···*A*
O1W—H1A···O2	0.890 (10)	1.929 (11)	2.811 (3)	171 (3)
O4—H4···O2^ii^	0.82	1.74	2.535 (2)	163
O1W—H1B···O3^iii^	0.889 (10)	2.177 (19)	2.934 (3)	143 (2)

Symmetry codes: (ii) *x*−1/2, *y*+1/2, *z*; (iii) −*x*, −*y*+1, −*z*.
